# Beyond NGS data sharing for plant ecological resilience and improvement of agronomic traits

**DOI:** 10.1038/s41597-024-03305-0

**Published:** 2024-05-08

**Authors:** Ji-Su Kwon, Jayabalan Shilpha, Junesung Lee, Seon-In Yeom

**Affiliations:** Department of Horticulture, Division of Applied Life Science (BK21 Four), Institute of Agriculture & Life Science, Gyeongsang National University, Jinju, 52828 Korea

## Abstract

Decoding complex plant omics is essential for advancing our understanding of plant biology, evolution, and breeding as well as for practical applications in agriculture, conservation, and biotechnology. The advent of Next-Generation Sequencing (NGS) has revolutionized global plant genomic research, offering high-throughput, cost-effective, and accurate methods for generating genomic data. However, challenges still exist that suggest an entirely unresolved genome characterized by high heterozygosity, extensive repetitive sequences, and complex ploidy features. In addition, individual investigation of genomic information from various genetic resources is essential for omics research, as there are differences in traits within a single breed beyond a species due to the uniqueness of sequence variation. This article provides high-quality genomic and transcriptomic insights targeted at the agronomical background.

Plants rely on complex defence mechanisms to endure environmental stresses, encompassing both abiotic and biotic factors. However, our understanding of these mechanisms is still lacking and incomplete. Genomic information serves as a fundamental element for studying crucial aspects of plant life such as adaptation to diverse environments. In this context, this collection presents high-quality genomic information for plants and other organisms of agricultural significance such as insect pests and fungal pathogens, which will become the basis for future agricultural industries. Furthermore, the collection described massive datasets related to genomics, transcriptomics, metagenomics, plant science, and plants of ecological or agricultural interest through multiplatform sequencing. Thus, the generated comprehensive integrated data enhances genome annotation by improving gene prediction, identifying non-coding elements, and characterizing regulatory regions.

The collection’s wide variety of species makes it useful for evolutionary and major trait analyses, as well as interspecies comparisons through in-depth variation analysis. Additionally, it can be used for functional genetics analyses to identify valuable traits associated with agriculture in various cultivation environments. Sequencing information to be used in the above research must have a high depth and coverage, be highly accurate, and use species that are close to the trait of interest and are highly important. The collection met these criteria and included species of high importance that foster advancements in crop improvement, evolutionary studies, and ecological resilience. This article highlights data processing and generation, utilizing multiplexed sequencing platforms to address challenges in comprehending plant ecology, promoting conservation efforts, and enhancing agronomic traits in important agronomic species.

The genome assemblies in this collection were mostly performed using long-read sequencing techniques, such as Pacific Bio Sciences HiFi and Oxford Nanopore sequencing. These methods have the advantage of reducing gaps in the assembly sequence by using long reads to connect the longer contigs, unlike the once common method of short-read genome assembly. However, long-read sequencing has a problem of low sequence accuracy compared to short reads, so it has mainly been used as a research method to supplement the polishing process through short read data^1^. The HiFi method included in this collection improves sequence accuracy, solving the major problems of long read data and enabling the construction of more accurate genomes. And, most studies in this collection utilized the Hi-C method to investigate the spatial organization of genomes, with two studies opting for the Omni-C method.

## The genomic data gives a greater understanding of agricultural interests in wild relatives beyond reference crops

Currently, high-quality PacBio HiFi reads were used to obtain chromosome-scale assemblies of wild species, including wild rice (*Oryza coarctata*)^2^, a relative of wild wheat, (*Aegilops umbellulate*)^3^, and a seasonal vegetable species, Zicaitai which belongs to the *Cruciferae* family^4^. The N50 of each study was found as 23.1 Mb, 17.7 Mb and 43.82 Mb, respectively, resulting in long contigs. These contigs were assigned to pseudochromosomes using Hi-C and Omni-C data.

The collection included the chromosome level genome assembly of two medicinal species such as *Adenosma buchneroides* and *Sophora flavescens*^5,6^. The chromosome-level de novo assembly of *A. buchneroides* yielded a 442.84 Mb genome by generating 90.60X depth and about 95.55% of the genome was successfully assigned to 14 chromosomes. For *S. flavescens*, the draft genome size was estimated to be around 2.08 Gb, with over 80% identified as Transposable Elements (TEs). The draft genome of *A. buchneroides* was constructed by de novo assembly using HiCanu and Hifiasm. The sequence accuracy of the assembly was then improved by polishing using NextPolish. For *S. flavescens*, error-correction of Nanopore raw reads was performed using Canu and FMLRC, and a draft genome was assembled using Canu. The lack of a reference genome has constrained the understanding of evolutionary histories and impeded conservation initiatives for these medicinal species. Thus, the newly generated reference genome is anticipated to facilitate an understanding of the genetic basis and evolutionary aspects of the biosynthesis of active compounds in these medicinal plant species.

*Lithocarpus polystachyus* Rehder, known as sweet tea, holds medical significance because its leaves contain a substantial concentration of dihydrochalcones (DHCs) and exhibit antioxidant properties. Intending to explore the biosynthetic pathway and regulatory mechanisms of DHC, this study^7^ marks the first chromosome-scale genome assembly of a Lithocarpus species, featuring a 952 Mb genome, a contig N50 of 21.4 Mb, and a BUSCO score of 98.6% completeness. This genome assembly allows for studying gene functions, regulatory mechanisms, and trait improvement in sweet tea. Lu *et al*.^8^ conducted a study focusing on the genome assembly of watershield (*Brasenia schreberi*), an aquatic plant, historically cultivated as a vegetable in East Asia. Employing PacBio long reads, Illumina short reads, and Hi-C sequencing techniques, they successfully assembled the 1170.4 Mb genome, with 93.6% of it anchored to 36 pseudochromosomes, thus representing significant advancements in understanding the genetic makeup of this endangered aquatic plant species. This comprehensive genome serves as a crucial asset for advancing research in conservation, evolution, and molecular breeding within the context of this valuable species.

Generating genomic information and deciphering genetic architecture are crucial for conserving and developing timber species, contributing to improved forest productivity, wood quality, resilience, and sustainability. The collection included chromosome scale genome assemblies of some commercial timber trees^9–11^. Sahu *et al*.^9,10^ employed the de novo assembly program, Supernova to assemble diploid genomes of commercial timber trees and Mahogany species, whereas Yang *et al*.^11^ utilized the genome assemblers NECAT and NextDenovo in Hongmu species. The genomic data obtained from these forest trees will be a valuable resource for studying essential traits associated with environmental adaptation, including wood density, growth rate, disease resistance, hardness, and fiber cell wall thickness.

The genus *Populus* provides essential resources for addressing global needs such as paper production, biofuels, timber, bioremediation, and animal feed. Through genome sequencing of *Populus davidiana*, a pivotal aspen species employing Hi-C scaffolding, a 408.1 Mb genome was obtained with 19 pseudochromosomes. The genome, which exhibited 98.3% completeness in the BUSCO assessment, contains 44.9% transposable elements (TEs), and a subsequent functional annotation predicted 31,862 protein-coding sequences. This study has the potential to enhance our understanding of evolutionary and functional genomics within the *Populus* genus^12^ and sets the stage for further investigations into the genetic basis of adaptation and speciation in forest tree species. In another study, the coloured calla lily, native to southern Africa and part of the *Zantedeschia* genus, had its high-quality chromosome-level genome generated, boasting a size of 1,154 Mb, 98.5% contig anchoring into 16 pseudochromosomes, and 60.18% identification of repetitive sequences. Functional annotations were also assigned to 95.1% of the predicted protein-coding genes, along with 469 miRNAs, 1,652 tRNAs, 10,033 rRNAs, and 1,677 snRNAs^13^. Particularly remarkable is the identification of Gypsy-type LTR retrotransposons as the primary factor causing significant genome size variation in Araceae species, which provides a foundation for further research into the evolutionary, ecological, and functional implications of genome size dynamics in this plant family.

The collection comprised the genome assembly of highly endangered and ornamental plant species. The genome of *Rhododendron viallii*, a plant species with extremely small populations (PSESP), was resolved at the haplotype level with two haploid genomes, sized at 532.73 Mb and 521.98 Mb^14^. This work is crucial for preserving and conserving such rare ornamental species. Equally important is the de novo genome assembly of *Hibiscus syriacus*, a flowering hexaploid species^15^. The researchers overcame the challenges associated with assembling complex polyploid genomes by employing a combination of PacBio Sequel and Nanopore technologies, along with a suite of genome assemblers like Canu, Flye, NECAT, and others. This approach underscores the efficiency and robustness of using long-read sequencing technologies in conjunction with diverse assemblers to enhance genome continuity in complex plant genomes.

Understanding the molecular traits of invasive species, pathogens, and pests is essential for developing effective strategies to regulate or mitigate their impact on agriculturally significant plant species^16,17^. The prickly nightshade, *Solanum rostratum* Dunal, recognized as an invasive weed with detrimental impacts on agriculture, ecology, and human health, has been sequenced, resulting in a high-quality chromosome-level genome of 1,154 Mb. The study also conducted comparative genomics to elucidate the phylogenetic relationship among Solanaceous species^18^. The first-time genome sequence of this invasive Solanaceae malignant weed, serves as a crucial resource for further elucidating the pathways and genes involved in its strong ecological adaptability and stress resistance. By revealing the genetic mechanisms underlying its invasive behaviour, researchers can develop strategies to manage and mitigate the negative effects of this weed on ecosystems and agricultural lands.

Utilizing Nanopore platforms for genome sequencing produced a genome assembly of 564.5 Mb for the tomato pinworm, *Tuta absoluta*, a destructive pest affecting tomato crops^19^. Despite a low-quality reference genome for this species, the authors utilized advanced sequencing technologies to produce a chromosome-level genome assembly, overcoming these limitations and offering a more comprehensive and precise depiction of the tomato pinworm’s genome. The availability of this superior genome can facilitate detailed examination of insect invasion, chromosome restructuring, evolutionary processes, and strategies for pest management. These studies highlight these species’ adaptive mechanisms and open new avenues for developing targeted interventions.

Moreover, the synteny study with silkworms and fall armyworms provided a comparative framework for studying genetic adaptations, and species divergence within this group of agricultural pests. The generation of a high-quality, chromatin-organized reference genome for *Fusarium proliferatum*, a soil-borne pathogen responsible for sudden decline syndrome (SDS) in date palms of UAE, was achieved through PacBio HiFi sequencing combined with Omni-C data^20^. The integration of PacBio HiFi and Omni-C data for identifying pathogenic genes is a testament to the critical role of advanced genomic research in plant pathology. It paves the way for more precise disease management strategies essential for sustaining agricultural productivity.

The incorporation of various sequencing platforms, including Illumina, PacBio HiFi, and Hi-C scaffolding has resulted in chromosome-level genome assembly for the oriental armyworm, *Mythimna separata* which significantly threatens agricultural production^21^. By employing diverse methods, this study advances the genome assembly of *M. separata*, ensuring improved quality and accuracy compared to previous researches. These advancements could enable deeper exploration into the molecular mechanisms linked to detoxification and host adaptation in *M. separata*, laying crucial theoretical foundations for the enhancement of management strategies. It offers valuable insights into detoxification and host adaptation mechanisms, which are key to developing sustainable pest management approaches.

Lee *et al*.^22^ proposed constructing a global co-expression network using various RNA-seq data for biological and abiotic stress responses. The method selects valuable genes through the interaction of RNA-seq samples with different processing methods and compares the global and common networks. This method has identified genes, NLR genes and transcription factors that respond to biotic and abiotic stresses, revealing potential stress-related genes for complex environmental conditions. Transcriptome research is vital for understanding how plants respond to stresses by identifying active genes in various conditions^23,24^. The innovative integrated network analysis strategy utilized in this study offers a valuable approach for comprehending gene regulation and can be used to enhance crop resilience against environmental stressors in various plant species.

**Fig. 1:**
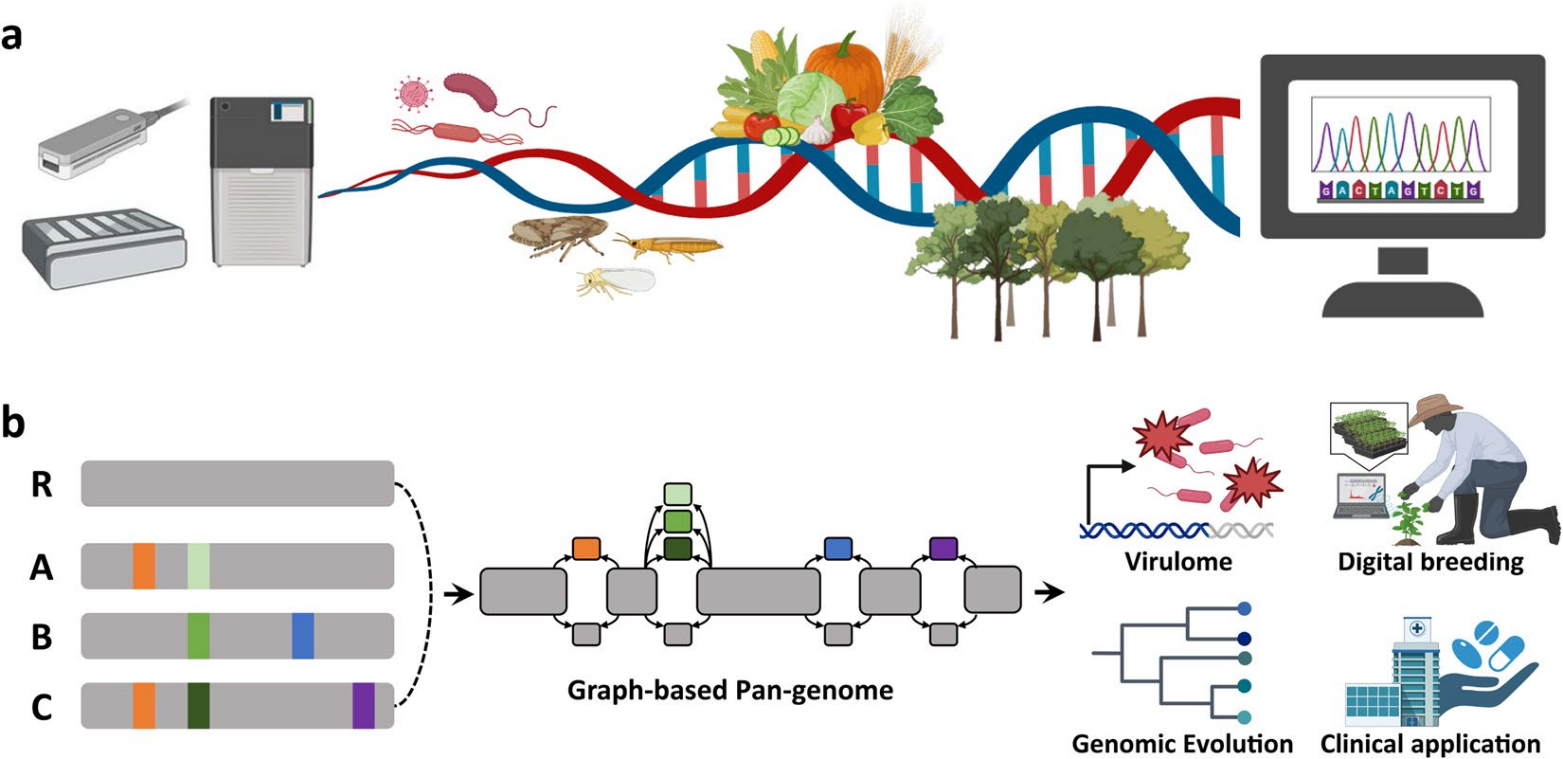
Integrative approaches in plant genomics and biotechnology for plant ecology, future agriculture. (**a**) Accumulation of various genomic/transcriptomic data related to agriculture. (**b**) Schematic representation of the application of pan-genome research in the agricultural field based on the accumulation of omics information. The gray long bars represent separate genomes, while the short, colorful bars indicate genes with variants.

One of the main points presented in this collection is that genomic information based on a variety of species forms the foundation of research, and, at the same time, it becomes possible to secure key genes based on transcriptomic information related to various environmental stresses (Fig. 1a). Consequently, this enables a cyclical research process where additional genetic information can be integrated into existing genomes. Therefore, the omics information for various organisms could complement each other, generating an efficient synergy in research.

## Advancing Plant Genomics: Chromosome-Level Assemblies and Pan-Genome Perspectives for Conservation, Breeding, and Disease Management

We made commentary about eighteen high-quality genomes at the chromosome level and one analysis article handling the transcriptome based on various stress conditions. The articles within this compilation represent a significant advancement in providing improved reference genomes of several endangered, medicinally and agronomically important plant species, pests, and pathogens. Besides, long-term monitoring of the genome assembly for potential updates or improvements based on emerging technologies or data could ensure the continuous improvement of reference genomes of these species.

Future research should focus on large-scale pan-genome analyses across diverse germplasm to capture a comprehensive representation of genetic variation and accelerate crop improvement through precision breeding strategies (Fig. 1b). This approach allows researchers to uncover additional genomic variations and understand the adaptive potential of a species in response to various environmental pressures. By incorporating pan-genome analysis into the current framework, researchers can not only provide a detailed understanding of the genetic makeup of these species but also elucidate how genomic variations contribute to traits such as stress tolerance, disease resistance, and medicinal properties.

Additionally, to maximize the impact of the genome assembly, these researches should consider establishing user-friendly online resources, such as a genome browser or data portal, to facilitate access and utilization of the genomic data by the broader scientific community. Also, establishing pan-genome databases alongside traditional genome browsers would facilitate comparative genomics and evolutionary studies across different populations or closely related species.

Addressing these criticisms and implementing the suggestions could strengthen the findings of this collection and contribute to a more comprehensive understanding of genetic and evolutionary features of these species. With the accumulation of data from diverse plant species through this collection and various genome assembly studies, coupled with advancements in bioinformatics technology, pan-genomics has emerged as a promising avenue for comprehending plant ecology, conservation, and agriculture.

Overall, the work presented in this collection not only enhances our comprehension of the genetic architecture of various plant and insect species but also underscores the importance of genomic research in addressing challenges in conservation, breeding, and agriculture. The continued advancement in this field promises a future where we can more effectively conserve biodiversity, improve crop yields, and manage pests and pathogens with greater precision and understanding.
